# AlGaN/AlN heterostructures: an emerging platform for integrated photonics

**DOI:** 10.1038/s44310-024-00048-z

**Published:** 2025-01-07

**Authors:** Sinan Gündoğdu, Sofia Pazzagli, Tommaso Pregnolato, Tim Kolbe, Sylvia Hagedorn, Markus Weyers, Tim Schröder

**Affiliations:** 1 Department of Physics, Humboldt-Universität zu Berlin, Berlin, Germany; 2 Ferdinand-Braun-Institut (FBH), Berlin, Germany

**Keywords:** Materials science, Materials for optics, Optics and photonics, Integrated optics

## Abstract

We introduce a novel material for integrated photonics and investigate aluminum gallium nitride (AlGaN) on aluminum nitride (AlN) templates as a platform for developing reconfigurable and on-chip nonlinear optical devices. AlGaN combines compatibility with standard photonic fabrication technologies and high electro-optic modulation capabilities with low loss over a broad spectral range, from UVC to long-wave infrared, making it a viable material for complex photonic applications. In this work, we design and grow AlGaN/AlN heterostructures and integrate several photonic components. In particular, we fabricate edge couplers, low-loss waveguides, directional couplers, and tunable high-quality factor ring resonators. These devices will enable nonlinear light-matter interaction and quantum functionality. The comprehensive platform we present in this work paves the way for photon-pair generation applications, on-chip quantum frequency conversion, and fast electro-optic modulation for switching and routing classical and quantum light fields.

## Introduction

**Fig. 1:**
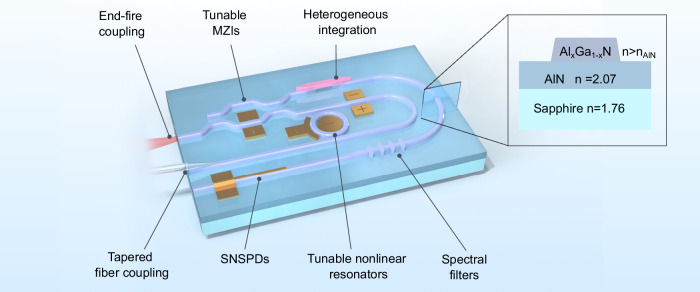
Conceptual illustration of the proposed AlGaN/AlN platform for photonic integrated circuits. A comprehensive AlGaN/AlN platform consists of a large variety of passive and active components. Here, we focus on passive and thermo-optically controlled devices. The core innovation of the material heterosystem is the photonic layer in which light is guided by engineering a few hundred-nanometer thick high refractive index top layer. This layer removes the optical mode from the sapphire substrate interface to reduce interface losses. Through lateral patterning, we integrate a large variety of optical elements, including waveguides, directional couplers, Mach-Zehnder interferometers, and thermo-optically tunable microring resonators as spectral filters. The integration of photonic crystals, electro-optic components, and superconducting nanowire single-photon detectors can in principle be realized but is beyond the scope of this work. Additionally, the AlGaN/AlN platform can in the future enable the heterogeneous integration of functional classical and quantum components such as laser diodes and solid-state quantum light sources, for example, defects in diamond and fluorescent molecules in organic matrices.

Advanced classical and quantum photonic applications, such as photonic neuromorphic computing^1^, quantum sensing^2^ and quantum networking^3–7^ rely on photonic integrated circuits that enable the compact, efficient, and high-rate implementation of a variety of optical functionalities. In addition to functionalities adapted from stand-alone optical devices, integrated circuits provide access to mode-multiplexing or routing^8,9^ and efficient fiber coupling. A comprehensive photonic device platform^10^, therefore, integrates various components, such as low-loss waveguides, efficient directional couplers, spectral filters, and tunable Mach-Zehnder interferometers (Fig. 1). A tunable ring resonator is another particularly versatile component, which can be employed to spectrally filter different modes, to enhance light-matter interaction, or to enable nonlinear frequency conversion and photon-pair generation^11–13^. Additionally, given the large variety of electronic and optical components readily available on different material platforms, like detectors^14,15^ or quantum light sources^16,17^, heterogenous integration is a key approach for building photonic integrated chips with a high level of complexity. Therefore, an ideal photonic platform should facilitate these methods while exhibiting properties such as low optical losses, fast electro-optic modulation, and significant optical nonlinearities to route and manipulate the propagating photons efficiently.

Different material platforms are currently being studied, each chosen for specific applications based on their distinct features. Lithium niobate on insulator (LNOI) is one of the leading platforms for devices requiring high optical nonlinearities with an outstanding electro-optic coefficient^18–21^. Silicon nitride (SiN) is a well-established material platform known for its low losses, particularly in the infrared range^22^. It is also compatible with standard silicon manufacturing processes and heterogeneous integration of active and passive materials^23^. SiN has demonstrated impressively low losses in the visible spectrum, ranging from 0.01 dB/cm to 0.09 dB/cm at wavelengths of 461 nm and 802 nm^24^. Although SiN is a centrosymmetric material with an intrinsically weak electro-optic effect, it can exhibit second-order nonlinearities (*χ*^(2)^ = 0.2 pm/V) due to the photogalvanic effect, which is characterized by slow time dynamics^25^. Another nitride-based material, aluminum nitride (AlN) on sapphire, is currently being explored due to its substantial nonlinear coefficient, its broad transparency range, and its moderate electro-optic coefficient^26–30^.

In this work, we introduce a platform complementary to AlN and based on aluminum gallium nitride (AlGaN), which offers a unique combination of properties: flexibility in epitaxial growth, transparency over a wide range of wavelengths, especially in the UV spectral range, compatibility with other GaN devices such as lasers and transistors, and the possibility of doping to achieve conductivity. AlN and GaN also have high thermal conductivity, which is ideal for high-power applications. We compare the key properties of LNOI, SiNx, and AlN/AlGaN/GaN photonic platforms in Table 1.

**Table 1:** Properties and technologies associated with LiNbO_3_, SiN_*x*_, and AlN/AlGaN/GaN for integrated photonics applications

	LNOI	SiN_*x*_	AlN/AlGaN/GaN
Band gap wavelength	329 nm^61^	234–273 nm^62^	199 nm (AlN) to 352 nm (GaN)^63^
*χ*^(2)^ nonlinearity	${\chi }_{33}^{(2)}$ = −83.4 pm/V at 1058 nm^64^	*χ*^(2)^ = 0.2 pm/V (photogalvanically induced) at 1557 nm^25^	${\chi }_{33}^{(2)}$ = −6.4 pm/V (*x* = 0.42) 3.9 pm/V (*x* = 0.66)^58^
Electro-optic coefficient	r_13_ = 9.6 pm/V r_22_ = 6.8 pm/V r_33_ = 30.9 pm/V at 500 nm^64^	Intrinsically small due to its centrosymmetry	r_13_ = 0.49 pm/V r_33_ = 0.78 pm/V *x* = 0.45 at 633 nm^65^
Growth technology	Smart-cut	PECVD, LPCVD or sputtering	Sputtering and MOVPE
Highlights	High nonlinear and electro-optic coefficients	Ultra-low loss, CMOS-compatible	Moderate nonlinearity, Heterostructure growth, GaN compatible, Electrically conductive, High thermal conductivity

x is the mole fraction of the Al_*x*_Ga_1−*x*_N.

Specifically, the photonic platform we propose consists of an epitaxial AlGaN layer grown on AlN on sapphire which acts as a guiding photonic layer and that can be patterned into various optical components. This approach overcomes challenges in AlN on sapphire platforms, such as the growth of crystal defects at the sapphire-AlN interface and lateral thickness variation, and introduces further material-specific functionality. With the additional AlGaN layer, we reduce optical losses and improve device performance compared to AlN on sapphire by shifting the optical mode away from this interface. The ability to control the ratio of aluminum to gallium in the ternary alloy Al_*x*_Ga_1−*x*_N allows for tailored properties of the photonic layer, such as bandgap and refractive index engineering. Moreover, AlGaN’s wide band gap enables efficient light transmission across a broad spectrum, depending on composition from below 250 nm to long-wave infrared. Doping AlGaN for electrical conductivity combines optical and electronic functionalities, enabling, for example, current-induced refractive index change^31,32^.

Our AlGaN platform benefits from the advanced state of the art of GaN, which has, over the past decade, evolved into a foundational material for electronic and electro-optic devices, including power electronics and light generation^33,34^. For example, GaN LEDs and lasers are widely used for producing blue and green light^35,36^. The epitaxial growth of GaN/AlGaN/InGaN heterostructures on sapphire templates, a well-established practice, opens possibilities for integrating light sources and electronics on the photonic platform.

The inherent properties of AlGaN, such as its relatively high electro-optic coefficient, make it an ideal candidate for rapid modulation and the development of reconfigurable optical devices. Its significant second-order optical nonlinearity paves the way for on-chip nonlinear photonic devices, including parametric oscillators and sum or difference frequency generators. The generation of correlated photon pairs in the ultraviolet (UV) and visible spectrum has been proposed using an AlGaN/AlN integrated photonics platform via spontaneous four-wave mixing (SFWM) in an AlGaN microring resonator^37^. AlGaN has also been explored as a potential platform for stimulated Brillouin scattering devices such as a racetrack Brillouin laser^38^.

To the best of our knowledge, only very few studies have used AlGaN as a photonic material. Li et al.^39^ fabricated large, multimode waveguides with an AlN/GaN multiple quantum well core and Al_0.1_Ga_0.9_N claddings. Although this is a notable achievement, the large size and relatively high sidewall roughness do not enable application for single-mode photonics. In addition, the wavelength range is limited to around 350 nm and longer. Bruch et al.^40^ fabricated ring resonators on Al_0.1_Ga_0.9_N nanomembranes transferred onto SiO_2_/Si substrates. While their work demonstrated the versatility of AlGaN, using nanomembranes introduces difficulties with handling. Recently, Shin et al. showcased the enhancement of the Pockels effect in multiple AlGaN/AlN quantum wells on AlN due to the large built-in polarization of the quantum wells^41^. Despite encountering relatively high propagation losses (18.3 dB/cm), their work achieved a 20-fold increase in second-order susceptibility compared to bare AlN. This work shows the capability of the AlGaN/AlN as a platform for cutting-edge engineering to achieve nonlinearities beyond those available in bulk materials.

In the present work we focus on the fabrication of the passive devices, which constitute the fundamental building blocks of the photonic platform shown in Fig. 1: waveguides, directional couplers, ring resonators. To achieve this, we grow Al_*x*_Ga_1−*x*_N, with x = 0.69, directly on high-temperature annealed AlN-on-sapphire templates with a reduced dislocation density^42^. Finally, we present the design of a device to achieve efficient and flexible phase matching for entangled photon generation using spontaneous parametric down-conversion (SPDC) at telecom wavelengths employing such a heterostructure.

## Methods

### Wafer growth and characterization

**Fig. 2:**
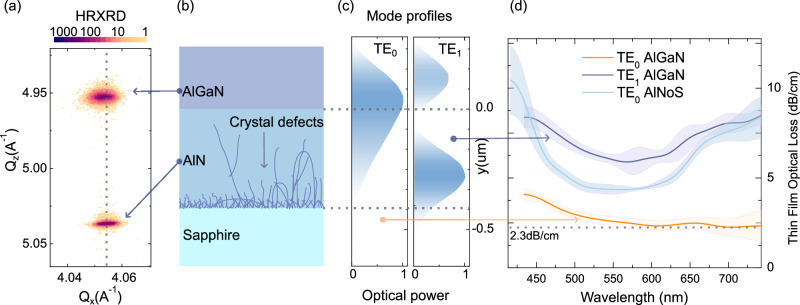
Material characterization. **a** High-resolution X-ray diffraction reciprocal space maps (11-24 reflection) illustrate the strain relaxation in the AlGaN and AlN layers. Two distinct peaks correspond to the AlN and AlGaN layers, respectively. Analysis of these peaks reveals an Al-mole fraction *x* = 0.69 and a mere 3% relaxation of in-plane compressive strain in the AlGaN layer, indicating a predominantly pseudomorphic growth on the AlN template and avoiding the generation of strain-relief defects. **b** Illustration of the basic heterostructure on a sapphire substrate. **c** Supported TE_0_ and TE_1_ modes for a 250 nm thick AlGaN layer on AlN at 632 nm. **d** The optical loss spectrum of these modes in the AlGaN/AlN heterostructure and the TE_0_ mode of a 0.4 μm thick AlN single layer on sapphire film (AlNoS), measured by prism coupling, where shaded areas indicate the uncertainty bounds.

The core ingredient of our novel integrated photonics platform is an AlGaN heterostructure (Fig. 2b). The AlN/AlGaN layer stack is epitaxially grown on a c-plane-oriented sapphire substrate with an offcut of 0.25° towards an m-plane. First, a 350 nm thick AlN layer is deposited using epitaxial magnetron sputtering. The sputtered material’s threading dislocation density (TDD) was decreased through high-temperature annealing (HTA), following the process described by Miyake et al.^43^. During HTA, a temperature of 1700 °C is maintained for a relatively short duration of 1h to prevent the formation of aluminum oxynitride on the AlN surface^44^. Subsequently, the rough sputtered and annealed surface is smoothened by MOVPE growth of 50 nm AlN in a step flow growth regime. The AlN layer, with a total thickness of 400 nm, exhibits a TDD of 7.5 × 10^8^ cm^−2^, as estimated from high-resolution X-ray diffraction (HRXRD) measurements of the symmetric (0002) and skew-symmetric (10–12) *ω*-rocking-curves^45^. Atomic force microscopy (AFM) revealed a surface RMS roughness of 0.09 nm over a 25 μm^2^ area.

Next, a 250 nm thick AlGaN waveguide layer is grown by MOVPE in a 6 × 2-inch close-coupled showerhead reactor on the AlN template, utilizing trimethylaluminum, triethylgallium, and ammonia as source materials, with hydrogen as the carrier gas, and a growth temperature of 1015 °C. In-situ reflectometry measurements verify the attainment of the targeted layer thickness of 250 nm. In-situ reflectometry measurements from the center and edge of the wafer have shown a deviation of 2 nm in the AlGaN layer thickness. After AlGaN growth, we measure the surface RMS roughness by AFM to be 0.5 nm. This smooth surface supports integrating photonic devices as introduced here and enables the hetero-integration of superconducting nanowire single-photon detectors (SNSPDs)^14^ and quantum devices^16^. The strain relaxation towards the underlying AlN buffer and the Al-mole fraction x of the Al_*x*_Ga_1−*x*_N layers is determined by HRXRD using *ω* − *ω*/2*Θ* reciprocal space maps (RSM) of the 11-24 reflection. Each RSM shows two sharp peaks (Fig. 2a), corresponding to the AlN template and AlGaN layers. By analyzing these data, an Al-mole fraction x of 0.69 and a relaxation of the in-plane compressive strain of only 3% can be determined for the AlGaN layer. This shows that the AlGaN grew almost pseudomorphically on the AlN template, and the generation of misfit dislocations for strain relief can thus be largely excluded.

### Optical material properties

We use the prism coupling technique as described in^46,47^ to quantify the optical losses in the as-grown thin films. It utilizes a rutile prism to couple a tunable white laser (SuperK Fianium) into the heterostructures. The losses are quantified by monitoring the decay of the scattered light with a digital camera. We characterize and compare a 400 nm AlNoS sample and an AlGaN/AlN/sapphire wafer. The AlNoS supports a single TE_0_ mode at 630 nm, whereas the AlGaN structure exhibits two distinct modes, TE_0_ and TE_1_, at the same wavelength. By fine-tuning the coupling angle, we isolate and analyze the losses of these modes, as reported in Fig. 2d.

The TE_0_ mode, in the AlGaN structure, exhibits optical losses that decrease with increasing wavelength, reaching a minimum value of 2.3 dB/cm above 600 nm. In contrast, the TE_1_ mode of the AlGaN and the TE_0_ mode of the AlNoS exhibited higher losses, with minimum values of approximately 5.9 and 4.3 dB/cm, respectively, which increase again for wavelengths over 625 nm. It is worth noting that both of these modes are localized at the interface between two different materials and, thus, are sensitive to the interface’s inherent properties. Besides the roughness at the interface, additional sources of loss could be due to defects and color centers in AlN, which are presumably concentrated at the AlN-sapphire interface as shown by TEM measurements by^44,48,49^. AlN has defects comprising a mix of impurities, vacancies, and lattice defects. Factors like threading dislocations influence the formation of these defects, although the exact conditions for their formation are yet not fully understood^50–52^. Our platform overcomes these material challenges by utilizing the AlGaN as a photonic waveguide layer.

### Device fabrication

**Fig. 3:**
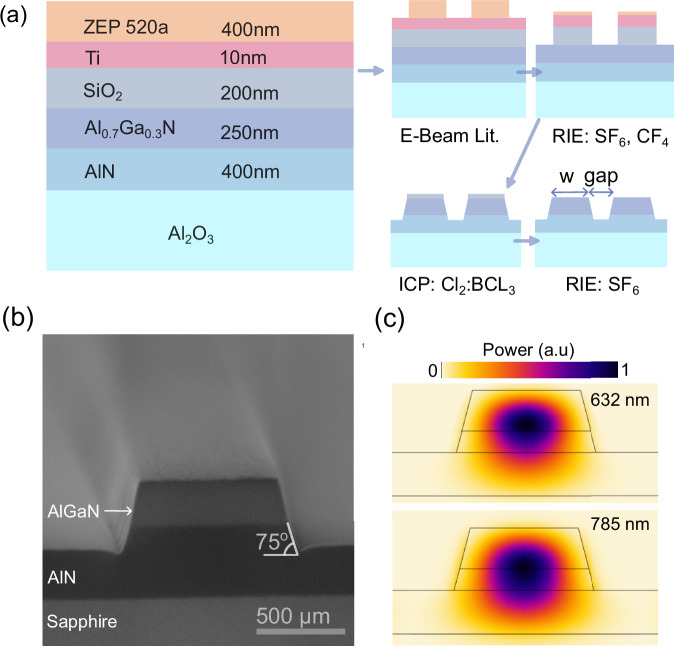
Fabrication geometries. **a** Epitaxial and mask layers for the fabrication of the optical nanowaveguides. **b** SEM image of a cleaved facet of a waveguide, where the different materials can be clearly identified. **c** Simulated optical power distribution of the fundamental TE mode of the waveguides at 632 nm and 785 nm.

In the fabrication process of waveguides and ring resonators, we initially deposit a 200 nm thick SiO_2_ layer using plasma enhanced chemical vapor deposition (PECVD) to serve as a hard mask. We then coat a 10 nm thick Ti layer via e-beam evaporation to act as a charge dissipation layer, followed by 400 nm ZEP 520A e-beam lithography resist. The process steps are illustrated in Fig. 3a. After patterning the resist, we transfer the pattern to the hard mask using a two-step reactive ion etching (RIE) process: we utilize SF_6_ plasma to etch Ti and CF_4_ plasma to etch SiO_2_. In the final etching step, we process the AlGaN layer using BCl_3_:Cl_2_:He at a ratio of 10:50:10 sccm, under 600 W ICP power and 100 W RF power, at a pressure of 1 Pa. The AlGaN-to-SiO_2_ etch rate ratio is approximately 3/1. The etch rate of AlGaN and AlN is similar, and our device designs work optimally when AlN is also etched. Therefore, after AlGaN is etched, we continue to etch into AlN 70 nm deep. Instead of utilizing an etch stop layer, we monitor the etch rate and depth using an in-situ interferometer. An etch-stop layer is a feasible future method; however, it requires additional materials in the growth process and, hence, additional growth recipe optimization. We implement a laser-cut notch at the chip’s edge to facilitate precise cleaving. Figure 3b presents an SEM image of a cleaved facet, revealing the AlN and AlGaN layers. We measure the waveguides to have a wall angle of 75°. Figure 3c shows the simulated power profiles of the fundamental TE mode of the waveguides at wavelengths of 632 nm and 785 nm. At the measurement wavelength of 785 nm used for our ring resonators, 90% of the optical power is located above the AlN-sapphire interface. At the shorter wavelength of 632 nm, this number increases to 95%, indicating that most of the optical power is well separated from the lossy boundary at the sapphire interface.

### Optical charactherization

**Fig. 4:**
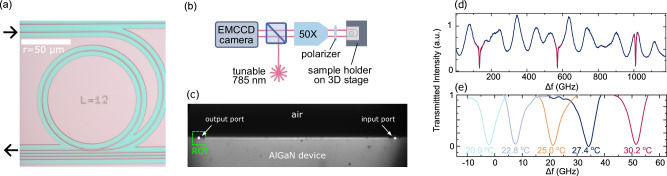
Ring resonator and waveguide device characterization. Optical microscope image of a waveguide coupled ring resonator (**a**) and a sketch of the experimental setup used for its characterization (**b**). The sample is mounted vertically and aligned through a 3D stage so that both the input and output ports can be imaged within the same field of view (**c**). The laser is focused on the input port and its frequency is scanned. During this process, a region of interest (ROI) around the output port is continuously monitored and recorded. Time is converted to frequency steps and the average intensity at the output port is plotted against frequency. **d** Typical transmission signal through a ring resonator (in this case with a width of 580 nm and a 100 nm coupling gap) plotted as a function of laser detuning. The dark red lines represent fits using a Lorentzian function with a quadratic baseline. **e** The resonance frequency tuning with temperature.

We fabricate microring resonators with a radius *r* of 50 μm, top width of 580 nm, and coupled to racetrack single-mode waveguides with the same width and cleaved at both ends, as depicted in Fig. 4a. The sample is mounted on a 3D piezoelectric nanopositioner (Physik Instrumente, NanoCube) and characterized with a custom-made inverted microscope (in Fig. 4b), where the same objective (Zeiss, LD EC Epiplan-Neofluar, 20X, NA = 0.22) is used to launch the laser light into one waveguide facet and collect it from the other one (see Fig. 4c). The alignment of the incoupling light is monitored with an EMCCD camera (Andor, iXon Ultra 897) and optimized by maximizing the intensity of the outcoupled signal by adjusting both sample position and laser incoupling angle. The total propagation loss per unit length in the ring *α* is then inferred by measuring the intrinsic Q-factor *Q*_0_ of the microring resonators according to equation^29^, $\alpha =10\cdot \,\text{log(e)}\frac{{\lambda }_{0}}{{Q}_{0}\cdot \text{FSR}\,\cdot r}$, with *λ*_0_ the resonant wavelength and FSR the free spectral range. To reach critical coupling we fabricate several microring resonators coupled to racetrack waveguides with varied coupling gaps, ranging from 100–325 nm. We measure the transmission of a tunable 785 nm laser (Toptica, DFB PRO centered at 784.6 nm, mode-hop-free over 2 nm) and use a linear film polarizer (Thorlabs, LPVIS) to excite and collect TE or TM modes selectively.

## Results

### Micro-ring resonators

Figure 4d shows the typical transmission as a function of laser frequency, where peaks corresponding to the same resonance and separated by the expected FSR can be observed above a baseline that is primarily due to interference in the measurement optics. This modulation can be described with a quadratic baseline from which the resonant peaks can be discriminated and fitted with a Lorentzian function, as indicated in red in Fig. 4d. With a 230 nm coupling gap, we observed near-critical coupling with a transmittance of 0.07 and an FWHM of 2.4 GHz for the TE mode, while the TM mode was under-coupled with a transmittance of 0.21 and an FWHM of 5.5 GHz. A racetrack geometry can be implemented to increase the coupling efficiency for the TM mode.

In order to test the temperature tunability of the TE and TM modes resonance frequencies, the chips are mounted on a custom-made temperature-controlled stage capable of heating the sample in the range of 20–30 °C. Figure 4e plots the shift of a resonant peak of the TE mode (coupling gap = 100 nm) for different temperatures. In the investigated temperature range, the central frequency of the resonance changes linearly with temperature, with coefficients of 5.7 and 6.2 GHz/K for TE and TM modes, respectively. These values are comparable to previous reports for AlN (2.94 × 10^−5^ K^−1^) and GaN (7.01 × 10^−5^ K^−1^)^53^ at 785 nm and 300 K and correspond to the thermo-optic coefficient for AlGaN.

Key parameters such as the Q-factors, the propagation losses, and the thermo-optic coefficients for TM and TE modes are summarized in Table 2.

**Table 2:** Summary of the measured and calculated optical and thermo-optic parameters at 785 nm for the Al_*x*_Ga_1−*x*_N (*x* = 0.69) ring resonators with 50 μm radius and 230 nm coupling gap

Parameter	TE	TM
FWHM (GHz)	2.4	5.5
FSR (GHz)	438	426
Propagation loss (dB/cm)	2.4	-
Q factor	1.6 × 10^5^ (critical)	0.7 × 10^5^ (undercoupled)
*dν*/*dT* at 785 nm (GHz/K)	5.7	6.2
*dn*/*dT* (×10^−5^ K^−1^)	2.9	3.26

### Directional couplers

**Fig. 5:**
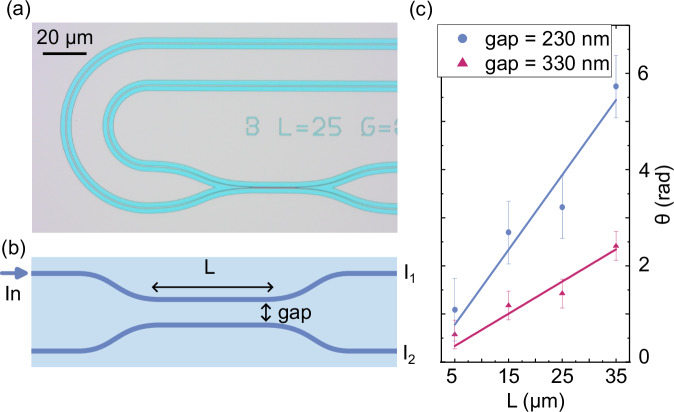
Directional couplers. **a** Optical microscope image of an AlGaN directional coupler. **b** Schematic illustration of the geometry, where the relevant parameters are presented. **c** Output intensity ratios across various coupling lengths and gap sizes, enabling the determination of the coupling constant for gaps measuring 230 and 330 nm.

Directional couplers are essential components in photonic integrated circuits. They enable the transfer of electromagnetic energy between two or more waveguides that are in proximity. They can be combined to form optical switches or Mach-Zehnder interferometers, which are the fundamental constituents of most on-chip quantum technology applications^54,55^. In our study, we develop AlGaN directional couplers linked to racetrack waveguides, as depicted in Fig. 5a. We vary coupling gaps and lengths, as illustrated in Fig. 5b. By injecting a 632 nm laser into one of the input ports, we measure the output intensity ratio: $\theta ={\tan }^{-1}({I}_{1}/{I}_{2})$. Our observations reveal a direct linear correlation between the coupling length and *θ*, with linear fitting of the data providing the coupling constants. For coupling gaps of 230 nm and 330 nm, the coupling constants were determined to be 0.15 μm^−1^ and 0.066 μm^−1^, respectively. The uncertainties observed are mainly due to reflections at the input and output facets and possible variations in the mirror reflectivity at the output waveguide facets. Additionally, variations in edge coupling losses can result from device geometry, alignment errors, and mismatches between the laser spot and the guided mode, which are influenced by the optical setup. Light is coupled into one port of the directional coupler to mitigate these effects and improve measurement accuracy, and the intensity ratio of the outputs is measured. This method ensures that the ratio remains independent of the input coupling efficiency. However, reflections can still impact the measurements as the reflected laser can be observed at the second input of the directional coupler. These reflections can be minimized by applying an anti-reflection coating. Further improvement is to be expected by optimizing the etching processes towards vertical sidewalls.

### Phase matching for nonlinear frequency conversion and pair generation

**Fig. 6:**
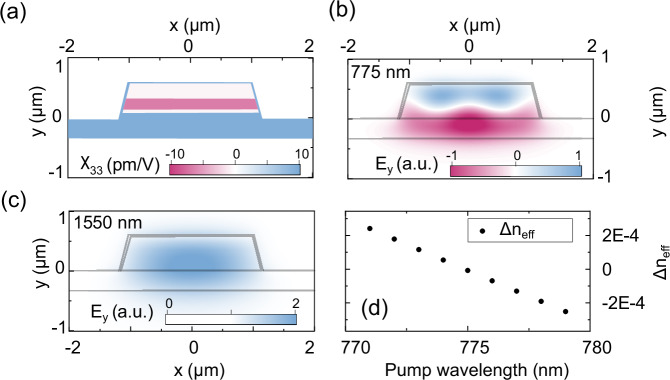
Spontaneous parametric down converison (SPDC). An optimized heterostructure of Al_*x*_Ga_1−*x*_N for entangled photon pair generation at 1550 nm. **a** Second-order nonlinear susceptibility tensor element *χ*_33_, **b** vertical component of the electric field of the higher order TM mode at 775 nm, **c** the fundamental TM mode at 1550 nm, and **d** effective index difference between these two modes as a function of pump laser wavelength.

In nonlinear photonics applications, achieving a phase matching of the modes involving the nonlinear conversion process is critical for efficiency. For integrated photonic waveguides, a common strategy is to match the effective indices of two distinct modes, typically a fundamental mode and a higher order mode^37,56^. These approaches, however, suffer from a weak overlap between the involved modes. The mode overlap integral for second harmonic generation and SPDC for degenerate photon pairs is defined as^57^

1 $$\Gamma =\left\vert \int\,{\chi }^{(2)}:{{\bf{E}}}_{{\boldsymbol{\omega }}}^{\,{2}}{{\bf{E}}}_{{\bf{2}}\omega }^{\,{*}}\,{\rm{d}}\Omega \right\vert ,$$

where *χ*^(2)^ is the second-order nonlinear susceptibility tensor, **E**_**ω**_ and **E**_**2ω**_ are the normalized mode electric field amplitudes of the modes at *ω* and 2*ω*, respectively. The integral is calculated over the mode volume *Ω*. In the case of phase-matched TM modes for AlGaN waveguides, the dominating term in the overlap integral is

2 $$\Gamma =\left\vert \int\,{\chi }_{33}{E}_{1y}^{2}{E}_{2y}^{\,{*}}{\rm{d}}x\,{\rm{d}}y\right\vert ,$$

where *χ*_33_ is the relevant component of *χ*^(2)^ for the interaction along the primary axis of the crystal in TM modes, and *E*_1*y*_ and *E*_2*y*_ are the y-components of the electric field amplitudes. Al_*x*_Ga_1−*x*_N alloys exhibit a sign change of the second-order nonlinear coefficient tensor component (*χ*_33_) at an alloy content of approximately *x* = 0.65^58^. We use this property to enhance the mode overlap. We design a heterostructure to achieve type-0 phase matching between the fundamental TM mode at 1550 nm and a higher-order mode at 775 nm with identical polarization, as demonstrated in Fig. 6a–c. This strategy facilitates efficient phase matching, enabled by the sign change of *χ*_33_, which significantly increases the overlap integral between the pump and SPDC modes. Our concept also allows for fine-tuning phase matching by adjusting the pump wavelength. The resultant effective index difference, *Δn*_*eff*_, between the pump and down-conversion modes, which converge to zero at approximately 775 nm, is depicted in Fig. 6d. For this simulation, we used a finite element solver (Comsol Multiphysics) to calculate the modes’ effective indices and used a genetic algorithm (supplied by Matlab) to minimize the objective function, (10^2^*Δn*_*eff*_ − *Γ*). From bottom to top, optimized AlGaN layer thicknesses are 110/70/290/50 nm on 400 nm AlN on sapphire, while the alloy compositions are 0.55/0.42/0.65/0.66. The waveguide’s top width is 1.98 μm, and the AlN layer is over-etched to a depth of 60 nm. Additionally, we define a 30 nm conformal AlN cladding. This particular layer design will likely exhibit some degree of strain relaxation, especially in the layers with low Al content. Changes in strain and piezoelectric polarization may impact the alignment of this calculation with experimental results. However, the overall viability of this method remains unaffected. The AlGaN/AlN heterostructure concept enables the engineering of compact SPDC sources and phase-matched ring resonators across a wide spectral range when coupled to a single-mode waveguide in a ring waveguide.

## Discussion

We present a new material platform based on vertical AlGaN heterostructures, which can be used to implement photonic devices with the core functionalities required to develop complex photonic integrated chips. By epitaxially growing AlGaN on HTA-AlN templates on sapphire substrates, we alleviated the challenges related to crystal defects and dislocations at the sapphire-AlN interface and achieved an RMS surface roughness as low as 0.5 nm. The optical losses we quantify, 2.5 dB/cm for TE_0_ mode and 7.1 dB/cm for TM_0_ mode at 785 nm, outperform earlier measurements on aluminum nitride on sapphire waveguides in the visible spectrum. For instance, Lu et al. found losses of 5.3 dB/cm at 633 nm for the TE_0_ mode^59^. It is worth noting that we did not use any cladding, which could reduce scattering losses, as one of our main interests is the heterogeneous integration of diamond-based photonic elements such as^16,17^ for which the cladding represents a physical obstacle for evanescent coupling. We chose the 400 nm AlN layer due to the same design, which is optimal for this thickness. The layers could be grown up to several (>5) micrometers thicknesses by extending the MOVPE AlN overgrowth. However, due to the high-temperature annealing applied to our AlN/sapphire template, no significant decrease in defect density, particularly in threading dislocation density, is achieved with thicker AlN layers. Thicker layers result in a stronger wafer bow, which can make processing more challenging. In our case, losses could be reduced by optimizing the layer sequence, growth recipes (e.g., by annealing the wafer after AlGaN growth), and device fabrication.

By measuring the thermo-optic coefficient and anisotropic refractive indices of the waveguides, we have gained insight into parameters relevant to applications such as nonlinear photon-pair generation and on-chip nonlinear conversion. The birefringent refractive index of our devices opens avenues for exploration, especially in its utilization for phase matching of TE and TM modes. Furthermore, the surface roughness of the epitaxial material is sufficiently low to integrate superconducting single-photon detectors^60^.

Our work introduces AlGaN heterostructures as a novel platform for integrated photonics. It combines broadband optical transparency, flexibility of epitaxy, and moderate optical nonlinearity, complementing well-established materials and filling an important niche in the field.

## Data Availability

The datasets used and/or analysed during the current study are available from the corresponding author on reasonable request.
